# Quality of Life after Deep Brain Stimulation in Parkinson's Disease: Does the Target Matter?

**DOI:** 10.1002/mdc3.14199

**Published:** 2024-09-03

**Authors:** Sandra Murcia Carretero, Katrin Petermann, Ines Debove, Deborah Amstutz, Mário Sousa, Julia Waskönig, Andreas Antonios Diamantaras, Gerd Tinkhauser, Andreas Nowacki, Claudio Pollo, Michael Schuepbach, Paul Krack, Martin Lenard Lachenmayer

**Affiliations:** ^1^ Department of Neurology Bern University Hospital and University of Bern Bern Switzerland; ^2^ Graduate School for Health Sciences University of Bern Bern Switzerland; ^3^ Department of Neurosurgery Bern University Hospital and University of Bern Bern Switzerland; ^4^ Institute of Neurology Konolfingen Switzerland

**Keywords:** Parkinson's disease, subthalamic nucleus, globus pallidus, deep brain stimulation, quality of life

## Abstract

**Background:**

Deep brain stimulation (DBS) of the subthalamic nucleus (STN) and globus pallidus internus (GPi) is an accepted therapy for Parkinson's disease (PD) with disabling motor complications. For elderly patients with poorer cognition and postural instability, GPi has been proposed as the preferable DBS target based on expert opinion, arguing GPi‐DBS may be less complicated by depression, apathy, worsened verbal fluency, and executive dysfunction, resulting in greater improvement in quality of life (QoL). However, data supporting such patient‐tailored approach are lacking.

**Objectives:**

The aims were to analyze whether the DBS target influences QoL in a PD cohort and a matched subgroup of frail patients with poor cognitive status and reduced postural stability, and whether other factors affect the QoL outcomes.

**Methods:**

In this retrospective study, we analyzed a single‐center cohort of 138 PD patients who received bilateral STN‐DBS (117) or GPi‐DBS (21) using the mentioned approach for target selection. All patients underwent standardized clinical evaluations of motor‐ and nonmotor signs as well as QoL before and 1 year after surgery.

**Results:**

DBS of both targets improved motor signs, dyskinesias, and pain. QoL improved without significant difference between the targets, but with a trend for greater improvement across all QoL domains in favor of the STN, even in an STN subgroup matched to the GPi group.

**Conclusion:**

Our results contradict the prevailing belief that GPi‐DBS is superior in frail PD patients with cognitive decline and postural instability, questioning the proposed patient‐tailored approach of DBS target selection. Further studies are needed for a data‐driven approach.

Parkinson's disease (PD) is a neurodegenerative disorder characterized by motor and nonmotor signs with a profound impact on quality of life (QoL).^1^, ^2^ Deep brain stimulation (DBS) of the subthalamic nucleus (STN) or the globus pallidus internus (GPi) is a well‐established therapy for levodopa (l‐dopa)‐responsive PD with disabling motor complications and is able to improve motor^3^ and nonmotor symptoms^4^ as well as QoL.^5^, ^6^

Although STN may be the first choice as a DBS target in many centers, as reflected by the striking numerical predominance of STN‐DBS studies over GPi‐DBS studies^7^ and the belief that STN target leads to better motor outcomes,^3^, ^8^, ^9^ there is an ongoing debate about the optimal DBS target for PD.^3^ The results of the only 2 randomized controlled trials (RCT) directly comparing these 2 targets did not provide conclusive evidence to support the superiority of either GPi‐DBS or STN‐DBS,^10^, ^11^ although 1 of these trials^11^ demonstrated greater improvement in motor signs and activities of daily living (ADL) in *off*‐medication phase with STN‐DBS than with GPi‐DBS. As a result, there is still a transatlantic divide on this issue, which can be explained in particular by differences between the European and North American health‐care systems.^12^ However, the interpretation of the secondary outcomes of the U.S. Veterans Administration study^10^ led to the promotion of a patient‐tailored DBS target selection favoring the GPi in case of cognitive or behavioral issues.^13^, ^14^

In the following years, many experts chose the STN target in younger PD patients, whereas the GPi target became the preferred DBS target for elderly PD patients who were frailer with poorer cognitive function, higher neuropsychiatric burden, or prominent gait and axial impairment.^15^

Nevertheless, studies confirming the superiority of GPi‐DBS applying such target selection criteria are lacking.^3^ Therefore, the current retrospective single‐center study aimed to determine STN‐DBS and GPi‐DBS outcomes with a particular focus on the QoL in a real‐world PD cohort that received target selection according to the proposed patient‐tailored approach.

## Patients and Methods

Standard protocol approvals, registrations, and patient consent were obtained. The Swiss Ethics approved the study (KEK no.: 2020‐02392).

### Study Population

We retrospectively identified patients diagnosed with Parkinson's disease who underwent bilateral STN‐DBS or GPi‐DBS implantation at the University Hospital of Bern between 2009 and 2018. The time window was defined based on the period in which the target selection was performed according to the patient‐tailored approach similar to that proposed by Williams et al.^15^ General selection criteria for DBS included advanced PD, good response to l‐dopa, disabling motor fluctuations refractory to medication and/or l‐dopa‐induced dyskinesia despite the best medical treatment, and the absence of surgical or medical contraindications, including major depression with suicidal thoughts, dementia, or acute psychosis. Advanced age, impaired postural stability, and mild cognitive impairment were individual features favoring the GPi target selection over STN. The choice of target was made during a multidisciplinary DBS board, taking into account the patient's motor, cognitive, and mood profiles, as well as the patients' own expectations.^15^

All patients underwent bilateral DBS surgery, which was performed as previously reported.^16^ The correct position of the electrodes was verified during awake surgery using electrophysiology. Microelectrode recording was used to identify the well‐known electrophysiological target signatures, and macrostimulation was used to assess intraoperative improvement in rigidity and absence of pyramidal diffusion.

All patients underwent postoperative axial computed tomography or magnetic resonance imaging (MRI) within 1 week after surgery to exclude surgical complications. To determine the correct electrode position, the postoperative imaging was systematically fused with the preoperative T2‐weighted MRI.

Postoperatively, stimulation settings and antiparkinsonian medication were gradually adapted based on the patient's need and clinical response. We included all patients who had completed the 39‐item Parkinson's Disease Questionnaire (PDQ‐39) to assess self‐reported QoL at both preoperative and postoperative assessments 1 year after surgery.

### Clinical Measures

All patients underwent standardized clinical evaluations before and 1 year after surgery. These assessments included the PDQ‐39^17^, ^18^ as the main outcome parameter as well as Part III of the Movement Disorder Society Unified Parkinson's Disease Rating Scale (MDS‐UPDRS‐III) or the Unified Parkinson's Disease Rating Scale (UPDRS‐III),^19^, ^20^ Schwab and England Activities of Daily Living,^21^ Marconi Dyskinesia Rating Scale,^22^ Mini‐Mental State Examination (MMSE),^23^ Hamilton Rating Scale for Depression (HAM‐D),^24^ Starkstein Apathy Scale,^25^ and Visual Analog Scale for Pain (VAS worst pain).^26^ Insomnia and postural stability subscores were extracted from the sum of items 4, 5, and 6 of the HAM‐D, and item 30 of the UPDRS‐III or 3.12 of the MDS‐UPDRS‐III, respectively. When only UPDRS‐III total scores were available, these were transformed into MDS‐UPDRS‐III scores, as described by Goetz et al.^27^ All scales were performed in “*on*‐medication” condition except MDS‐UPDRS‐III, which was performed in “*on*‐ and *off*‐medication” conditions before surgery and in “*off*‐medication/ON‐stimulation” and “*on*‐medication/ON‐stimulation” conditions 1 year postoperatively. The “*off*‐medication” condition was assessed after 48 h of withdrawal of dopamine agonists and an overnight withdrawal of l‐dopa medication. The best “*on*‐medication” condition was assessed after the administration of a rapid‐release l‐dopa formulation. Levodopa equivalent daily dose (LEDD) was calculated according to Tomlinson et al.^28^ and Schade et al.^29^

### Statistical Analysis

All statistical analyses were performed using R, version 4.2.2 (2022‐10‐31).^30^ The code is accessible on GitHub (https://github.com/kilyth/qol_dbs).

Missing variables except PDQ‐39 were imputed using a random forest algorithm provided in the *R* package *missForest*, version 1.5.^31^ This algorithm uses random forest trained on the observed clinical measures to predict the missing values. It can be used to impute continuous and categorical data simultaneously, including complex interactions and nonlinear relations between the variables.

Linear models were used to assess the relationship between different clinical factors and QoL. The signs of the differences between pre‐ and postoperative values were chosen such that a positive change indicates an improvement on the respective scale. Thus, positive coefficients resulting from the linear models for change variables indicate an improvement in QoL.

To quantify the relative contributions of the regressors to the linear model's total explanatory value, we calculated the relative importance metric pmvd (proportional marginal variance decomposition as proposed in Feldman^32^) using the *R* package *relaimpo*, version 2.2‐6.^33^

For a direct comparison of QoL outcome between targets, STN and GPi groups were matched based on preoperative clinical factors (Table 2), thereby minimizing the selection bias due to the clinical choice of the stimulation target. Propensity score matching was calculated using the *R* package *Matching*, version 4.10‐8,^34^ and the resulting difference in QoL was compared to 10^4 permutations to estimate the significance of the result.

Percentile bootstrap confidence intervals (CI) for the percentage change values were calculated using 10,000 samples and 0.025 and 0.975 quantiles of the resulting distribution. All results are given with their 95% CI or ±standard deviation.

Figures were created using the *R* package *ggplot2*, version 3.4.1.^35^

## Results

### Study Population

In total, the cohort included 202 PD patients who received bilateral STN‐DBS or GPi‐DBS at the University Hospital of Bern between 2009 and 2018. From these, 21 withdrew consent, 35 were lost to follow‐up, and 8 did not complete the PDQ‐39 at both visits. Finally, 138 patients were included in the analysis, of whom 117 received STN‐DBS and 21 GPi‐DBS. No serious intra‐ or postoperative complications with disabling long‐term side effects occurred.

As shown in Table 1, the 2 groups had different baseline characteristics, with patients in the GPi group being older and having a longer disease duration, worse QoL, less improvement in MDS‐UPDRS‐III in the l‐dopa challenge, higher dyskinesia scores, and worse postural stability. For the MMSE there was a statistically significant difference of <1 point. There were no significant differences regarding LEED, MDS‐UPDRS‐III in the “*off*‐medication” condition, ADL (Schwab and England Scale), insomnia, pain, or neuropsychiatric symptoms (Hamilton, Starkstein scales).

**TABLE 1:** Demographics and baseline characteristics (N = 138)

		STN	GPi	*P*‐value
N		117	21	
Gender (%)	Male	76 (65.0)	9 (42.9)	0.094
	Female	41 (35.0)	12 (57.1)	
Age at surgery		63.19 (8.90)	70.11 (6.77)	0.001
Disease duration (yr)		11.88 (4.32)	14.51 (4.75)	0.013
Time from surgery to assessment (weeks)		58.55 (13.47)	61.72 (21.56)	0.373
PDQ‐39 SI		28.51 (12.90)	34.52 (10.25)	0.045
LEDD		1246.15 (608.42)	1149.05 (483.28)	0.490
MDS‐UPDRS‐III (*off* medication)		41.83 (14.43)	41.76 (12.95)	0.984
MDS‐UPDRS‐III (% improvement)		60.62 (14.19)	54.67 (10.21)	0.069
Schwab and England		64.36 (17.86)	62.86 (13.09)	0.714
MMSE		28.62 (1.32)	27.38 (1.72)	<0.001
Hamilton		7.52 (5.32)	6.38 (4.68)	0.360
Starkstein		11.57 (5.35)	12.90 (5.26)	0.294
Pain		4.48 (3.22)	4.53 (2.89)	0.945
Marconi		6.44 (5.82)	9.00 (5.07)	0.061
Insomnia		1.85 (1.52)	1.76 (1.30)	0.793
Postural stability		0.74 (0.79)	1.14 (0.96)	0.041
Stimulator type (%)	Medtronic	67 (57.3)	16 (76.2)	0.165
	Boston	50 (42.7)	5 (23.8)	

*Note*: Continuous variables are summarized by mean and standard deviation (in parentheses), whereas the categorical variables are listed in counts and percentage (in parentheses).

Abbreviations: STN, subthalamic nucleus; GPI, globus pallidus internus; PDQ‐39 SI, Parkinson's Disease Questionnaire Summary Index; LEDD, levodopa equivalent daily dose; MDS‐UPDRS‐III, Movement Disorder Society Unified Parkinson's Disease Rating Scale, Part III; MMSE, Mini‐Mental State Examination.

In a subanalysis to compare the QoL between the GPi and STN groups, we used a propensity score–matched cohort to minimize selection bias. The matched cohort included 42 patients, 21 patients in the STN subgroup and 21 in the GPi group, who differed only by the target (Table 2).

**TABLE 2:** Baseline characteristics of the matched cohort

		STN subgroup	GPi	*P*‐value
N		21	21	
Gender (%)	Male	10 (47.6)	9 (42.9)	1.000
	Female	11 (52.4)	12 (57.1)	
Age at surgery		68.83 (5.25)	70.11 (6.77)	0.497
Disease duration (yr)		13.60 (3.86)	14.51 (4.75)	0.504
Time from surgery to assessment (weeks)		59.35 (16.77)	61.72 (21.56)	0.693
PDQ‐39 SI		34.19 (11.80)	34.52 (10.25)	0.923
LEDD		1096.67 (456.85)	1149.05 (483.28)	0.720
MDS‐UPDRS‐III (*off* medication)		42.90 (11.16)	41.76 (12.95)	0.761
MDS‐UPDRS‐III (% improvement)		56.19 (10.76)	54.67 (10.21)	0.640
Schwab and England		58.90 (17.38)	62.86 (13.09)	0.410
MMSE		27.86 (1.20)	27.38 (1.72)	0.303
Hamilton		8.24 (7.78)	6.38 (4.68)	0.354
Starkstein		14.38 (5.71)	12.90 (5.26)	0.389
Pain		4.77 (3.34)	4.53 (2.89)	0.802
Marconi		7.76 (5.73)	9.00 (5.07)	0.463
Insomnia		1.52 (1.50)	1.76 (1.30)	0.586
Postural stability		0.81 (0.60)	1.14 (0.96)	0.186
Stimulator type (%)	Medtronic	15 (71.4)	16 (76.2)	1.000
	Boston	6 (28.6)	5 (23.8)	

*Note*: Continuous variables are summarized by mean and standard deviation (in parentheses), whereas the categorical variables are listed in counts and percentage (in parentheses).

Abbreviations: STN, subthalamic nucleus; GPI, globus pallidus internus; PDQ‐39 SI, Parkinson's Disease Questionnaire Summary Index; LEDD, levodopa equivalent daily dose; MDS‐UPDRS‐III, Movement Disorder Society Unified Parkinson's Disease Rating Scale, Part III; MMSE, Mini‐Mental State Examination.

### QoL Outcome for the DBS Cohort: 1‐Year Follow‐Up

**FIG. 1:**
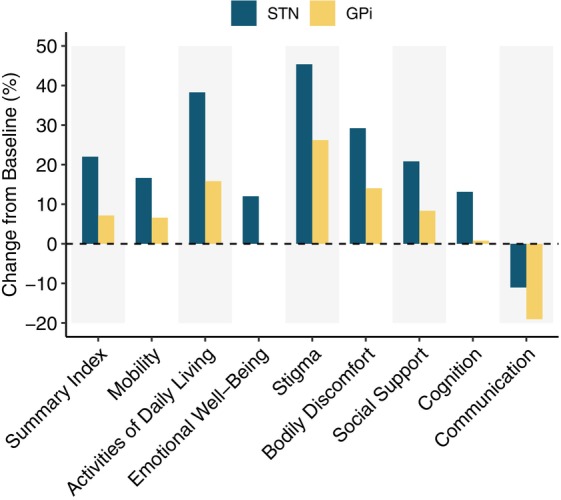
Mean percentage change before and 1 year after DBS (deep brain stimulation) implantation of PDQ‐39 SI (Parkinson's Disease Questionnaire Summary Index) and subscores. Results are shown for the whole cohort of 117 STN (blue) and 21 GPi patients (yellow). Positive values indicate an improvement in the respective subscore. GPi, globus pallidus internus; STN, subthalamic nucleus.

Overall, Parkinson's Disease Questionnaire Summary Index (PDQ‐39 SI) improved by 19.4% (CI: [9.68, 28.18]) 1 year postoperatively. Except for communication in both groups and emotional well‐being in the GPi group, all subscores improved, especially ADL, stigma, and bodily discomfort (Fig. 1). Although not statistically significant, we observed a trend toward higher improvement in both PDQ‐39 SI and all its respective subscores in the STN group compared to the GPi group. All other clinical outcome variables as well as stimulation parameters are shown in the Data S1 and in Figure S1, shown separately by target.

### Pre‐ and Postoperative Predictors of Improvement in QoL after DBS

Using multiple linear regression, age was the only preoperative predictor of QoL improvement at 1‐year follow‐up, showing on average a lower improvement in QoL at older ages (−0.27, CI: [−0.52, −0.01], *P*‐value = 0.039; Table S1).

**FIG. 2:**
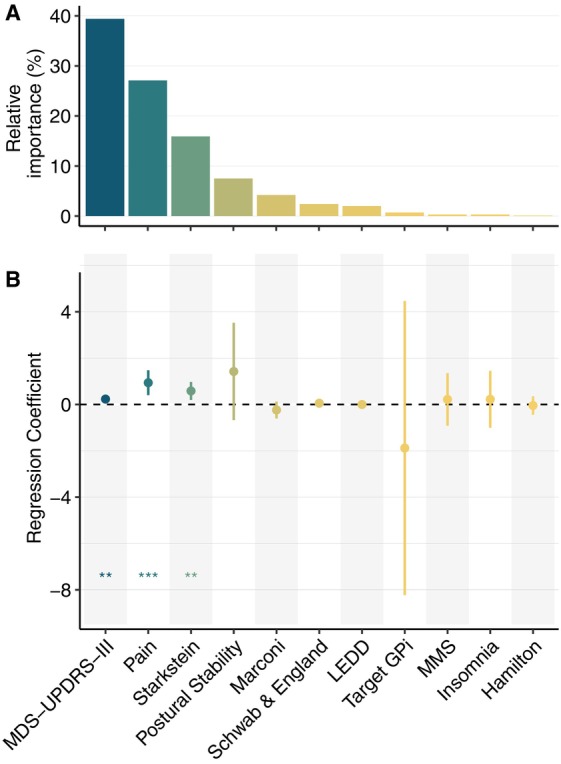
Results of the linear model showing the relationship between improvement in PDQ‐39 SI (Parkinson's Disease Questionnaire Summary Index) and improvement (or change in the target) in the respective variables. (**A**) Relative importance of the variables. (**B**) Regression coefficients with 95% CIs (confidence interval). For numeric results of the model see Table S2. GPI, globus pallidus internus; LEDD, levodopa equivalent daily dose; MMSE, Mini‐Mental State Examination; MDS‐UPDRS‐III, Movement Disorder Society Unified Parkinson's Disease Rating Scale, Part III.

To identify variables whose change after DBS influenced QoL, we performed a multivariable linear regression (Fig. 2) entering all factors modifiable by surgery as dependent variables. Improvement in motor outcome (MDS‐UPDRS‐III “*off* medication”) (0.23, CI: [0.09, 0.37], *P* = 0.001), reduction in pain (0.94, CI: [0.40, 1.47], *P* = 0.0007), and improvement in apathy (0.58, CI: [0.20, 0.97], *P* = 0.003) were factors that significantly contributed to improvement in QoL. Postural stability, dyskinesia, LEDD, ADL (Schwab and England), insomnia, cognition (MMSE), depression (Hamilton scale), and the target were not significantly related to change in PDQ‐39 SI.

### STN‐ versus GPi‐Matched Groups: 1‐Year Follow‐Up

Because the DBS target was chosen following a patient‐tailored approach, the lack of influence of the target on QoL could be due to selection bias. Using propensity score matching, we formed an STN subgroup, whose average baseline variables did not differ from the GPi cohort (Table 2). Comparing these 2 groups, we observed no significant differences on the postoperative PDQ‐39 SI but a tendency for STN patients to have a better QoL 1 year after surgery (4.3 ± 4.4 points on the PDQ‐39 SI, *P* = 0.17).

**FIG. 3:**
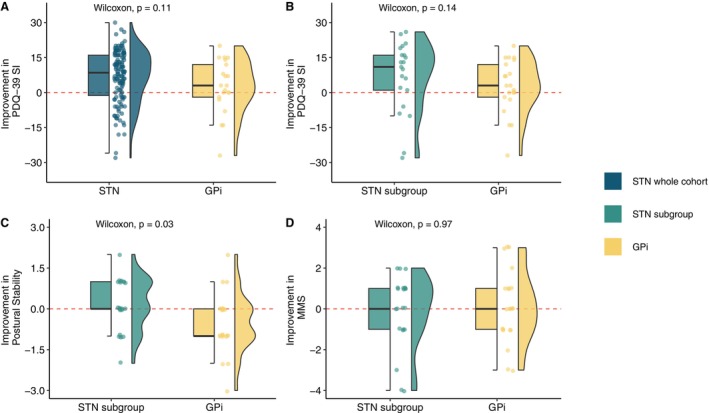
Absolute improvement in (**A**, **B**) PDQ‐39 SI, (**C**) postural stability, and (**D**) MMSE after DBS (deep brain stimulation). (A) Results for the whole cohort of 117 STN and 21 GPi patients. (B–D) Results of the 21 matched patients. GPI, globus pallidus internus; MMSE, Mini‐Mental State Examination; PDQ‐39 SI, Parkinson's Disease Questionnaire Summary Index; STN, subthalamic nucleus.

Postural stability and MMSE outcomes were important criteria for target selection. In the matched group, postural stability improved in the “*off*‐medication” condition in the STN subgroup (mean improvement: 0.19 ± 0.98), whereas in the GPi group it slightly worsened (mean change: −0.52 ± 1.12, *P*‐value = 0.03). There was no difference at 1 year postoperative in MMSE outcome between the 2 targets (Fig. 3).

## Discussion

In our retrospective analysis of a single‐center PD cohort with STN‐DBS or GPi‐DBS, we observed an average improvement in QoL of 19.4% measured by the PDQ‐39 (CI: [9.68, 28.18]) 1 year after DBS surgery, which is in line with previously reported findings from RCTs.^10^, ^11^, ^36^, ^37^ Notably, the improvement in QoL was not different between the STN and GPi cohorts, even after the clinical baseline profile of both target groups was matched. The findings of the present work therefore challenge the commonly proposed DBS target selection criteria preaching the noble art of patient‐tailored target selection by favoring the GPi in a frail population of PD patients with older age, poorer cognition, and postural instability.^14^, ^15^

Recently, Boogers and Fasano^12^ emphasized that the GPi should be more often reconsidered as a DBS target in PD and should be prioritized in elderly frail patients with poorer cognitive function, higher neuropsychiatric burden, or prominent gait and axial impairment. However, this recommendation is primarily based on individual experiences and the interpretation of the secondary outcomes from the U.S. Veterans Administration study,^10^ which demonstrated in a large neuropsychological assessment battery a significant but only slight decrease in processing speed and a minimal increase of 2/63 points on the Beck Depression Inventory‐II in the STN group compared to the GPi group, with no correction for multiple assessments. Studies justifying such a patient‐profile‐oriented DBS target selection are currently lacking. With overall heterogeneous and contradictory results in the literature, the question of the influence of the DBS target on QoL also remains unanswered. In contrast to our study, the few available comparative studies do not focus on frail PD patients and moreover provide contradictory results on QoL.^38^, ^39^ In addition, the short follow‐up period of 6 months^4^ could bias the results in favor of GPi‐DBS. Furthermore, studies with unilateral stimulation^38^, ^39^ do not provide any information that allows conclusions about the effect of bilateral DBS. Noteworthy, our analysis revealed a tendency for the STN‐DBS group to show a greater improvement after DBS in the overall PDQ‐39 and all its dimensions compared to the GPi‐DBS group. Nevertheless, the influence of the surgical target on QoL was not statistically significant, which might be explained by group differences due to our patient‐tailored approach with choosing the GPi as the DBS target for patients with older age, worse cognitive status, and poorer postural stability. However, since in our cohort the target selection was based on individual clinical judgment with many variables rather than using a strict selection algorithm, we identified a matched subgroup of older patients with worse cognitive scores and postural stability in the STN group that differed from the GPi‐DBS group only by target. Contrary to the expectation that the GPi target would be more beneficial in this subgroup of patients, there was no statistically significant difference in QoL improvement depending on the target. Instead, the trend of greater improvement persisted in the STN‐DBS subgroup. Whereas cognitive screening scores remained stable with no differences between the targets, postural stability in the “*off*‐medication” condition improved slightly in the STN subgroup and significantly worsened in the GPi group.

Regardless of the DBS target, our analysis further identified motor improvement in the “*off*‐medication” state, reduction in pain, and improvement in apathy as factors positively affecting QoL 1 year after DBS. Although numerous studies have already suggested a positive impact of motor improvement on QoL of PD patients after DBS,^40^, ^41^ current knowledge of PD‐related pain after DBS is limited. Nevertheless, and in line with our results, there is evidence for the overall efficacy of DBS in alleviating PD‐related pain,^42^, ^43^ with no significant differences observed between different DBS targets.^43^ Apathy, which is frequently identified as a determinant of QoL in PD,^2^ overall worsened after DBS in our cohort. Whereas for STN‐DBS this can be explained by the relevant reduction in dopaminergic medication,^44^ the increase in apathy among GPi‐DBS is surprising and might possibly be explained by an overlap with the observed worsening of depression scores. Nevertheless, our results highlight the impact of apathy on QoL after DBS and the importance of its early detection, as it can be improved by reintroducing or increasing dopamine agonists^45^, ^46^ provided that apathy is related to lack of motivation and not to dementia.^47^ Surprisingly, although depression has been identified as a significant predictor of QoL in PD,^1^, ^2^ it did not play a relevant role in the changes observed in our analysis. This discrepancy may be due to the fact that baseline depression scores and postoperative changes in depression were relatively low in both treatment groups, with no cases of severe depression as it is a contraindication for DBS in both targets. In the literature, postoperative changes in depression have been observed in both directions and are overall inconclusive. Moreover, the clinical relevance of very small changes reported in the past^10^ has probably been overestimated, as substantial changes are needed to affect QoL, which is a very robust measure. Thus, our study was likely underpowered to investigate such a hypothetical benefit.

Because QoL is a very complex construct, many other factors besides motor and nonmotor PD signs are likely to have an important impact on QoL, such as social support and family environment, individual expectations and coping strategies, patients’ cultural background, and distressing feelings such as shame. Tools and scales to measure these factors are needed to better understand and further improve the QoL of people with PD.

Our study has several limitations. As a retrospective study, data availability was limited; however, it is representative of real‐life outcomes, albeit from a single center with a long track of experience in both targets.^48^, ^49^ The small number of patients in the GPi group compared with the STN group may have an impact on our results. Furthermore, not all nonmotor signs were analyzed because of the lack of comparable scales in the assessments, parts I, II, and IV being hardly comparable between UPDRS and MDS‐UPDRS. For a differentiated and comparative assessment of cognition as an influential factor for QoL, the MMSE has limitations because it is a screening tool and is not very sensitive to detection of mild dysexecutive syndrome, which is typical for PD. The 21 STN patients matched to the GPi group indicated a good balance of clinical variables at baseline (Table 2). However, although this method accounts for known outcome parameters/covariates of analyses, thus minimizing selection bias, an imbalance of unknown parameters cannot be ruled out.

In conclusion, the analysis of our real‐life data confirms that both STN‐DBS and GPi‐DBS have a positive impact on QoL in PD. However, contrary to the prevailing belief that GPi should be the preferred target for older patients with worse cognitive status and poorer postural stability,^14^, ^15^ our analysis indicates that the GPi target is not superior to the STN target in improving QoL in this specific patient cohort. Moreover, considering our findings that motor improvement in the “*off*‐medication” condition is a factor that significantly affects QoL and that this improvement is even greater in STN‐DBS than in GPi‐DBS,^3^, ^11^ the STN might be the preferable DBS target even for patients with older age, worse cognitive status, and poorer postural stability. Although there are 10 times less published data on outcomes of GPi‐DBS compared to STN‐DBS in the classical indications,^3^ practically none exists for patients with borderline selection criteria concerning age, cognition, and l‐dopa‐resistant axial signs in either target, and indeed such patients have been excluded in past trials for good reasons.^50^, ^51^ Therefore, there is a need for prospective studies to explore the boundaries for indications, particularly because the current criteria for DBS target selection are based more on the interpretation of results and expert opinions^13^, ^14^ than on definitive study outcomes. Although this need exists, conducting such RCTs may be impractical given the existing studies comparing both DBS targets. Instead, it would be of great importance for clinical practice to develop a DBS registry to analyze and publish more retrospective data, which are readily available given the widespread use of DBS in recent decades with a substantial number of so‐called “frail” patients who have already undergone and continue to undergo DBS surgery. In this context, differences in health‐care systems and center experiences are also important to consider. Our results further suggest that in addition to the improvement in motor signs in the “*off* state,” change in pain and apathy are critical determinants of improvements in QoL after DBS and should be carefully and individually assessed. Identifying factors influencing QoL change after DBS may assist clinicians in focusing their assessments and treatment strategies to minimize PD‐associated disability.

## Author Roles

(1) Research project: A. Conception, B. Organization, C. Execution; (2) Statistical analysis: A. Design, B. Execution, C. Review and critique; (3) Manuscript preparation: A. Writing of the first draft, B. Review and critique.

S.M.C.: 1A, 1B, 1C, 2A, 2C, 3A

K.P.: 1A, 1B, 1C, 2A, 2B, 3B

I.D.: 1C, 2C, 3B

D.A.: 1B, 2C, 3B

M.S.: 1C, 2C, 3B

J.W.: 1C, 2C, 3B

A.‐A.D.: 1C, 2C, 3B

G.T.: 1C, 2C, 3B

A.N.: 1C, 2C, 3B

C.P.: 1C, 2C, 3B

M.S.: 1C, 2C, 3B

P.K.: 1A, 2C, 3B

M.L.L.: 1A, 1B, 1C, 2A, 2C, 3B

## Disclosures

**Ethical Compliance Statement**: This study was approved by the Swiss Ethics (Bern Cantonal Ethics Committee, KEK no.: 2020‐02392). Written informed consent for research was obtained from all individual participants involved in the study and documented in their electronic health records. We confirm that we have read the journal's position on issues involved in ethical publication and affirm that this work is consistent with those guidelines.

**Funding Sources and Conflicts of Interest**: No specific funding was received for this work. The authors declare that there are no conflicts of interest relevant to this work.

**Financial Disclosures for the Previous 12 Months**: Sandra Murcia Carretero reports having received a research grant from Boston Scientific outside of the submitted work. Ines Debove receives a research grant from Boston Scientific, as well as travel reimbursement and support for raising awareness in PD (video project) from Zambon, AbbVie, Boston Scientific, Bial, Ever, UCB, Medtronic, Spirig, Merz, and GE Healthcare. Ines Debove has also served on the advisory boards of Ever, Spirig, and AbbVie. All the mentioned entities provided support outside the scope of the submitted work. Gerd Tinkhauser received funding from the Swiss National Science Foundation (project number: PZ00P3_202166). He received financial support from Boston Scientific and Medtronic; he has a research agreement with RuneLabs and Medtronic not related to the present work. No specific funding was received for this work. Michael Schuepbach received funding from Research Form InnoMedica Switzerland and served as a consultant for Medtronic. No specific funding was received for this work. Paul Krack reports research or educational grants from the Swiss National Science Foundation (FNS 323530_177577/FNS 2020 32003BL_1 97709–1/FNS 33IC30_198772), ROGER DE SPOELBERCH Foundation, the Bertarelli Foundation, the Annemarie Opprecht Foundation, Parkinson Schweiz, the Michael J. Fox Foundation, Aleva Neurotherapeutics, Boston Scientific, Medtronic, GE Healthcare, and Idorsia; paid to employing institution and lecturing fees to employing institution from Boston Scientific, Bial, and Advisis; and travel expenses for scientific meetings from Boston Scientific, Zambon, and AbbVie. Martin Lenard Lachenmayer received a research grant from the Jacques und Gloria Gossweiler Foundation outside of the submitted work and has served on the advisory board for Bial.

## Supporting information


**Figure S1.** Percentage change before and 1 year after DBS (deep brain stimulation). Results are shown for the whole cohort of 117 STN and 21 GPi patients. Positive values indicate an improvement in the respective score. Single dots, result for each patient; bars, mean percentage change. GPI, globus pallidus internus; LEDD, levodopa equivalent daily dose; MDS‐UPDRS‐III, Movement Disorder Society Unified Parkinson's Disease Rating Scale, Part III; MMSE, Mini‐Mental State Examination; PDQ‐39 SI, Parkinson's Disease Questionnaire Summary Index; STN, subthalamic nucleus.

**Table S1.** Results of the linear model showing the relationship between the improvement in PDQ‐39 SI (Parkinson's Disease Questionnaire Summary Index) and the preoperative variables. Positive regression coefficients indicate that an increase in the respective variable leads to an improvement in QoL (quality of life). Residual standard error: 11.19 on 122 degrees of freedom (DF), adjusted *R* ^2^: 0.051, F‐statistic: 1.492 on 15 and 122 DF, *P*‐value: 0.11822. GPI, globus pallidus internus; LEDD, levodopa equivalent daily dose; MDS‐UPDRS‐III, Movement Disorder Society Unified Parkinson's Disease Rating Scale, Part III; MMSE, Mini‐Mental State Examination; STN, subthalamic nucleus; VAS, Visual Analog Scale for Pain.

**Table S2.** Results of the linear model showing the relationship between the improvement in PDQ‐39 SI (Parkinson's Disease Questionnaire Summary Index) and the improvement (or change in the target) in the respective variables. Positive regression coefficients indicate that an improvement in the respective variable leads to an improvement in QoL (quality of life). Residual standard error: 10.46 on 126 degrees of freedom (DF), adjusted *R* ^2^: 0.198, F‐statistic: 4.075 on 11 and 126 DF, *P*‐value <0.001. GPI, globus pallidus internus; LEDD, levodopa equivalent daily dose; MDS‐UPDRS‐III, Movement Disorder Society Unified Parkinson's Disease Rating Scale, Part III; MMSE, Mini‐Mental State Examination; STN, subthalamic nucleus.

**Data S1.** Clinical outcome variables and average stimulation parameters for the Parkinson's disease (PD) cohort.

## Data Availability

The data that support the findings of this study are available from the corresponding author upon reasonable request.

## References

[1] Schrag A , Jahanshahi M , Quinn N . What contributes to quality of life in patients with Parkinson's disease? J Neurol Neurosurg Psychiatry 2000;69(3):308–312. 10945804 10.1136/jnnp.69.3.308 PMC1737100

[2] Martinez‐Martin P , Rodriguez‐Blazquez C , Kurtis MM , Chaudhuri KR . The impact of non‐motor symptoms on health‐related quality of life of patients with Parkinson's disease. Mov Disord 2011;26(3):399–406. 21264941 10.1002/mds.23462

[3] Lachenmayer ML , Mürset M , Antih N , et al. Subthalamic and pallidal deep brain stimulation for Parkinson's disease‐meta‐analysis of outcomes. NPJ Parkinsons Dis 2021;7(1):77. 34489472 10.1038/s41531-021-00223-5 PMC8421387

[4] Dafsari HS , Dos Santos Ghilardi MG , Visser‐Vandewalle V , et al. Beneficial nonmotor effects of subthalamic and pallidal neurostimulation in Parkinson's disease. Brain Stimul 2020;13(6):1697–1705. 33038595 10.1016/j.brs.2020.09.019

[5] Dafsari HS , Weiß L , Silverdale M , et al. Short‐term quality of life after subthalamic stimulation depends on non‐motor symptoms in Parkinson's disease. Brain Stimul 2018;11(4):867–874. 29655587 10.1016/j.brs.2018.02.015

[6] Schuepbach WMM , Tonder L , Schnitzler A , et al. Quality of life predicts outcome of deep brain stimulation in early Parkinson disease. Neurology 2019;92(10):e1109–e1120. 30737338 10.1212/WNL.0000000000007037 PMC6442017

[7] Deuschl G , Antonini A , Costa J , et al. European academy of neurology/Movement Disorder Society‐European section guideline on the treatment of Parkinson's disease: I. Invasive therapies. Eur J Neurol 2022;29(9):2580–2595. 35791766 10.1111/ene.15386

[8] Krack P , Volkmann J , Tinkhauser G , Deuschl G . Deep brain stimulation in movement disorders: from experimental surgery to evidence‐based therapy. Mov Disord 2019;34(12):1795–1810. 31580535 10.1002/mds.27860

[9] Obeso JA , Olanow CW , Rodriguez‐Oroz MC , Krack P , Kumar R , Lang AE . Deep‐brain stimulation of the subthalamic nucleus or the pars interna of the globus pallidus in Parkinson's disease. N Engl J Med 2001;345(13):956–963. 11575287 10.1056/NEJMoa000827

[10] Follett KA , Weaver FM , Stern M , et al. Pallidal versus subthalamic deep‐brain stimulation for Parkinson's disease. N Engl J Med 2010;362(22):2077–2091. 20519680 10.1056/NEJMoa0907083

[11] Odekerken VJ , van Laar T , Staal MJ , et al. Subthalamic nucleus versus globus pallidus bilateral deep brain stimulation for advanced Parkinson's disease (NSTAPS study): a randomised controlled trial. Lancet Neurol 2013;12(1):37–44. 23168021 10.1016/S1474-4422(12)70264-8

[12] Boogers A , Fasano A . A transatlantic viewpoint on the role of pallidal stimulation for Parkinson's disease. Mov Disord 2024;39(1):36–39. 37965914 10.1002/mds.29656

[13] Okun MS , Foote KD . Parkinson's disease DBS: what, when, who and why? The time has come to tailor DBS targets. Expert Rev Neurother 2010;10(12):1847–1857. 21384698 10.1586/ern.10.156 PMC3076937

[14] Bronstein JM , Tagliati M , Alterman RL , et al. Deep brain stimulation for Parkinson disease: an expert consensus and review of key issues. Arch Neurol 2011;68(2):165. 20937936 10.1001/archneurol.2010.260 PMC4523130

[15] Williams NR , Foote KD , Okun MS . STN vs. GPi deep brain stimulation: translating the rematch into clinical practice. Mov Disord Clin Pract 2014;1(1):24–35. 24779023 10.1002/mdc3.12004 PMC4000041

[16] Nowacki A , Debove I , Fiechter M , et al. Targeting accuracy of the subthalamic nucleus in deep brain stimulation surgery: comparison between 3 T T2‐weighted magnetic resonance imaging and microelectrode recording results. Oper Neurosurg (Hagerstown) 2018;15(1):66–71. 28973406 10.1093/ons/opx175

[17] Fitzpatrick R , Peto V , Jenkinson C , Greenhall R , Hyman N . Health‐related quality of life in Parkinson's disease: a study of outpatient clinic attenders. Mov Disord 1997;12(6):916–922. 9399215 10.1002/mds.870120613

[18] Martinez‐Martin P , Jeukens‐Visser M , Lyons KE , et al. Health‐related quality‐of‐life scales in Parkinson's disease: critique and recommendations. Mov Disord 2011;26(13):2371–2380. 21735480 10.1002/mds.23834

[19] Fahn SER . Members of the UPDRS development committee. Unified Parkinson's disease rating scale. Recent Dev Parkinsons Dis 1987;2:293–304.

[20] Goetz CG , Tilley BC , Shaftman SR , et al. Movement Disorder Society‐sponsored revision of the unified Parkinson's disease rating scale (MDS‐UPDRS): scale presentation and clinimetric testing results. Mov Disord 2008;23(15):2129–2170. 19025984 10.1002/mds.22340

[21] Lang A . Clinical rating scales and videotape analysis. In: Koller WCPG , ed. Therapy of Parkinson's Disease. New York: Marcel Dekker; 1990:3–30.

[22] Marconi R , Lefebvre‐Caparros D , Bonnet AM , Vidailhet M , Dubois B , Agid Y . Levodopa‐induced dyskinesias in Parkinson's disease phenomenology and pathophysiology. Mov Disord 1994;9(1):2–12. 8139601 10.1002/mds.870090103

[23] Folstein MF , Folstein SE , McHugh PR . "mini‐mental state". A practical method for grading the cognitive state of patients for the clinician. J Psychiatr Res 1975;12(3):189–198. 1202204 10.1016/0022-3956(75)90026-6

[24] Hamilton M . A rating scale for depression. J Neurol Neurosurg Psychiatry 1960;23(1):56–62. 14399272 10.1136/jnnp.23.1.56 PMC495331

[25] Starkstein SE , Mayberg HS , Preziosi TJ , Andrezejewski P , Leiguarda R , Robinson RG . Reliability, validity, and clinical correlates of apathy in Parkinson's disease. J Neuropsychiatry Clin Neurosci 1992;4(2):134–139. 1627973 10.1176/jnp.4.2.134

[26] Price DD , McGrath PA , Rafii A , Buckingham B . The validation of visual analogue scales as ratio scale measures for chronic and experimental pain. Pain 1983;17(1):45–56. 6226917 10.1016/0304-3959(83)90126-4

[27] Goetz CG , Stebbins GT , Tilley BC . Calibration of unified Parkinson's disease rating scale scores to Movement Disorder Society‐unified Parkinson's disease rating scale scores. Mov Disord 2012;27(10):1239–1242. 22886777 10.1002/mds.25122

[28] Tomlinson CL , Stowe R , Patel S , Rick C , Gray R , Clarke CE . Systematic review of levodopa dose equivalency reporting in Parkinson's disease. Mov Disord 2010;25(15):2649–2653. 21069833 10.1002/mds.23429

[29] Schade S , Mollenhauer B , Trenkwalder C . Levodopa equivalent dose conversion factors: an updated proposal including Opicapone and safinamide. Mov Disord Clin Pract 2020;7(3):343–345. 32258239 10.1002/mdc3.12921 PMC7111582

[30] R Core Team . R: A Language and Environment for Statistical Computing. Vienna, Austria: R Foundation for Statistical Computing; 2022 https://www.R-project.org/.

[31] Stekhoven DJ , Bühlmann P . MissForest–non‐parametric missing value imputation for mixed‐type data. Bioinformatics 2012;28(1):112–118. 22039212 10.1093/bioinformatics/btr597

[32] Feldman BE . Relative importance and value SSRN; 2005. 10.2139/ssrn.2255827.

[33] Groemping U . Relative importance for linear regression in R: the package relaimpo. J Stat Soft 2006;17(1):1–27.

[34] Sekhon JS , Grieve RD . A matching method for improving covariate balance in cost‐effectiveness analyses. Health Econ 2012;21(6):695–714. 21633989 10.1002/hec.1748

[35] Wickham H . ggplot2: Elegant Graphics for Data Analysis. New York: Springer‐Verlag; 2016 https://ggplot2.tidyverse.org.

[36] Deuschl G , Schade‐Brittinger C , Krack P , et al. A randomized trial of deep‐brain stimulation for Parkinson's disease. N Engl J Med 2006;355(9):896–908. 16943402 10.1056/NEJMoa060281

[37] Weaver FM , Follett KA , Stern M , et al. Randomized trial of deep brain stimulation for Parkinson disease: thirty‐six‐month outcomes. Neurology 2012;79(1):55–65. 22722632 10.1212/WNL.0b013e31825dcdc1 PMC3385495

[38] Cernera S , Eisinger RS , Wong JK , et al. Long‐term Parkinson's disease quality of life after staged DBS: STN vs GPi and first vs second lead. NPJ Parkinsons Dis 2020;6:13. 32656315 10.1038/s41531-020-0115-3 PMC7338364

[39] Zahodne LB , Okun MS , Foote KD , et al. Greater improvement in quality of life following unilateral deep brain stimulation surgery in the globus pallidus as compared to the subthalamic nucleus. J Neurol 2009;256(8):1321–1329. 19363633 10.1007/s00415-009-5121-7 PMC3045861

[40] Daniels C , Krack P , Volkmann J , et al. Is improvement in the quality of life after subthalamic nucleus stimulation in Parkinson's disease predictable? Mov Disord 2011;26(14):2516–2521. 22170276 10.1002/mds.23907

[41] Sobstyl M , Ząbek M , Górecki W , Mossakowski Z . Quality of life in advanced Parkinson's disease after bilateral subthalamic stimulation: 2 years follow‐up study. Clin Neurol Neurosurg 2014;124:161–165. 25051167 10.1016/j.clineuro.2014.06.019

[42] Flouty O , Yamamoto K , Germann J , et al. Idiopathic Parkinson's disease and chronic pain in the era of deep brain stimulation: a systematic review and meta‐analysis. J Neurosurg 2022;137(6):1821–1830. 35535836 10.3171/2022.2.JNS212561

[43] Gong S , Xu M , Tao Y , et al. Comparison of subthalamic nucleus and Globus pallidus internus deep brain stimulation surgery on Parkinson disease‐related pain. World Neurosurg 2020;135:e94–e99. 31733388 10.1016/j.wneu.2019.11.026

[44] Lhommée E , Klinger H , Thobois S , et al. Subthalamic stimulation in Parkinson's disease: restoring the balance of motivated behaviours. Brain 2012;135(Pt 5):1463–1477. 22508959 10.1093/brain/aws078

[45] Martinez‐Fernandez R , Pelissier P , Quesada JL , et al. Postoperative apathy can neutralise benefits in quality of life after subthalamic stimulation for Parkinson's disease. J Neurol Neurosurg Psychiatry 2016;87(3):311–318. 25934016 10.1136/jnnp-2014-310189

[46] Thobois S , Lhommée E , Klinger H , et al. Parkinsonian apathy responds to dopaminergic stimulation of D2/D3 receptors with piribedil. Brain 2013;136(Pt 5):1568–1577. 23543483 10.1093/brain/awt067

[47] Béreau M , Kibleur A , Servant M , et al. Motivational and cognitive predictors of apathy after subthalamic nucleus stimulation in Parkinson's disease. Brain 2024;147(2):472–485. 37787488 10.1093/brain/awad324

[48] Lachenmayer ML , Bettschen C , Bernasconi C , et al. Stimulation of the globus pallidus internus in the treatment of Parkinson's disease: long‐term results of a monocentric cohort. Parkinsonism Relat Disord 2019;64:118–123. 30935828 10.1016/j.parkreldis.2019.03.009

[49] Debove I , Petermann K , Nowacki A , et al. Deep brain stimulation: when to test directional? Mov Disord Clin Pract 2023;10(3):434–439. 36949800 10.1002/mdc3.13667 PMC10026308

[50] Ory‐Magne F , Brefel‐Courbon C , Simonetta‐Moreau M , et al. Does ageing influence deep brain stimulation outcomes in Parkinson's disease? Mov Disord 2007;22(10):1457–1463. 17516457 10.1002/mds.21547

[51] Witt K , Daniels C , Krack P , et al. Negative impact of borderline global cognitive scores on quality of life after subthalamic nucleus stimulation in Parkinson's disease. J Neurol Sci 2011;310(1–2):261–266. 21733529 10.1016/j.jns.2011.06.028

