# Supplementary figures and images for: Quality of Life after Deep Brain Stimulation in Parkinson's Disease: Does the Target Matter?
Source: Mov Disord Clin Pract. 2024 Sep 3;11(11):1379–87. doi: 10.1002/mdc3.14199 (PMC11542293; doi:10.1002/mdc3.14199)

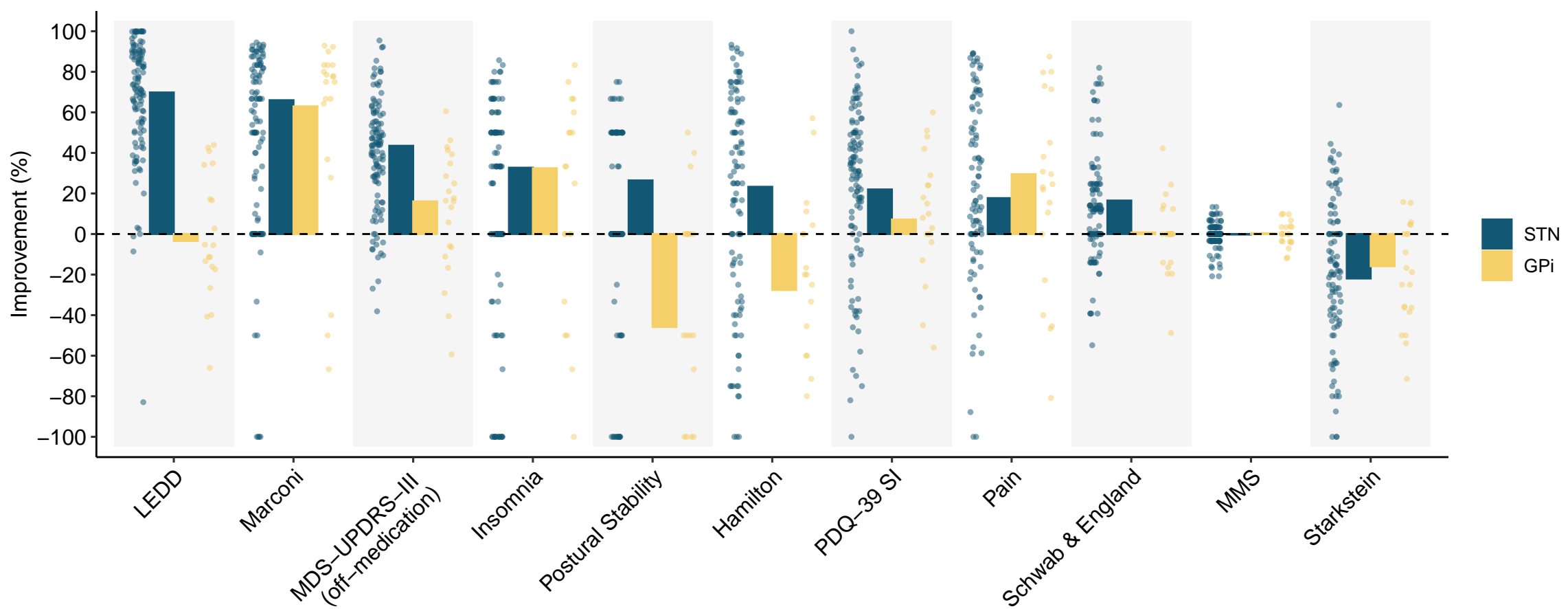

Supplement: Supplementary file 1 — Figure S1. Percentage change before and 1 year after DBS (deep brain stimulation). Results are shown for the whole cohort of 117 STN and 21 GPi patients. Positive values indicate an improvement in the respective score. Single dots, result for each patient; bars, mean percentage change. GPI, globus pallidus internus; LEDD, levodopa equivalent daily dose; MDS‐UPDRS‐III, Movement Disorder Society Unified Parkinson's Disease Rating Scale, Part III; MMSE, Mini‐Mental State Examination; PDQ‐39 SI, Parkinson's Disease Questionnaire Summary Index; STN, subthalamic nucleus. [file MDC3-11-1379-s001.pdf]
