# Supplementary material for: Quality of Life after Deep Brain Stimulation in Parkinson's Disease: Does the Target Matter?
Source: Mov Disord Clin Pract. 2024 Sep 3;11(11):1379–87. doi: 10.1002/mdc3.14199 (PMC11542293; doi:10.1002/mdc3.14199)
Supplement: Supplementary file 2 — Table S1. Results of the linear model showing the relationship between the improvement in PDQ‐39 SI (Parkinson's Disease Questionnaire Summary Index) and the preoperative variables. Positive regression coefficients indicate that an increase in the respective variable leads to an improvement in QoL (quality of life). Residual standard error: 11.19 on 122 degrees of freedom (DF), adjusted R 2: 0.051, F‐statistic: 1.492 on 15 and 122 DF, P‐value: 0.11822. GPI, globus pallidus internus; LEDD, levodopa equivalent daily dose; MDS‐UPDRS‐III, Movement Disorder Society Unified Parkinson's Disease Rating Scale, Part III; MMSE, Mini‐Mental State Examination; STN, subthalamic nucleus; VAS, Visual Analog Scale for Pain. [file MDC3-11-1379-s002.docx]

**Supplementary table 1**

|  | **Coefficient** | **95% confidence interval** | **p-value** | **Relative importance (%)** |
| --- | --- | --- | --- | --- |
| **Intercept** | 7.17 | from -44.04 to 58.38 | 0.78 |  |
| **Age at surgery** | -0.27 | from -0.52 to -0.01 | 0.039 | 17 |
| **Postural Stability** | 2.03 | from -0.72 to 4.79 | 0.15 | 13.4 |
| **MDS-UPDRS-III**  **(off-medication)** | 0.11 | from -0.06 to 0.27 | 0.20 | 12.1 |
| **Gender female** | 3.41 | from -1.26 to 8.08 | 0.15 | 10.8 |
| **Starkstein** | 0.30 | from -0.11 to 0.71 | 0.15 | 8.5 |
| **Marconi** | -0.33 | from -0.73 to 0.08 | 0.11 | 8.3 |
| **Target GPi** | -3.76 | from -10.19 to 2.68 | 0.25 | 7.3 |
| **Hamilton** | 0.15 | from -0.34 to 0.63 | 0.55 | 5.3 |
| **Disease duration (years)** | 0.32 | from -0.20 to 0.83 | 0.23 | 4.9 |
| **LEDD** | -0.0024 | from -0.01 to 0.00 | 0.21 | 4.4 |
| **VAS worst pain** | 0.32 | from -0.40 to 1.04 | 0.38 | 3.6 |
| **Insomnia** | 0.37 | from -1.34 to 2.08 | 0.67 | 2.5 |
| **MDS-UPDRS-III**  **(% improvement)** | 0.034 | from -0.13 to 0.19 | 0.68 | 1.1 |
| **Schwab & England** | 0.032 | from -0.11 to 0.17 | 0.65 | 0.8 |
| **MMS** | -0.028 | from -1.60 to 1.54 | 0.97 | 0 |
