# Supplementary material for: Quality of Life after Deep Brain Stimulation in Parkinson's Disease: Does the Target Matter?
Source: Mov Disord Clin Pract. 2024 Sep 3;11(11):1379–87. doi: 10.1002/mdc3.14199 (PMC11542293; doi:10.1002/mdc3.14199)
Supplement: Supplementary file 3 — Table S2. Results of the linear model showing the relationship between the improvement in PDQ‐39 SI (Parkinson's Disease Questionnaire Summary Index) and the improvement (or change in the target) in the respective variables. Positive regression coefficients indicate that an improvement in the respective variable leads to an improvement in QoL (quality of life). Residual standard error: 10.46 on 126 degrees of freedom (DF), adjusted R 2: 0.198, F‐statistic: 4.075 on 11 and 126 DF, P‐value <0.001. GPI, globus pallidus internus; LEDD, levodopa equivalent daily dose; MDS‐UPDRS‐III, Movement Disorder Society Unified Parkinson's Disease Rating Scale, Part III; MMSE, Mini‐Mental State Examination; STN, subthalamic nucleus. [file MDC3-11-1379-s004.docx]

**Supplementary table 2**

|  | **Coefficient** | **95% confidence interval** | **p-value** | **Relative importance (%)** |
| --- | --- | --- | --- | --- |
| **Intercept** | 4.51 | from 0.08 to 8.94 | 0.046 |  |
| **MDS-UPDRS-III** | 0.23 | from 0.09 to 0.37 | 0.001 | 39.4 |
| **Pain** | 0.94 | from 0.40 to 1.47 | 0.0007 | 27.1 |
| **Starkstein** | 0.58 | from 0.20 to 0.97 | 0.003 | 15.9 |
| **Postural Stability** | 1.42 | from -0.68 to 3.52 | 0.18 | 7.5 |
| **Marconi** | -0.24 | from -0.60 to 0.13 | 0.20 | 4.2 |
| **Schwab & England** | 0.055 | from -0.06 to 0.17 | 0.35 | 2.4 |
| **LEDD** | -0.0019 | from -0.01 to 0.00 | 0.27 | 2.0 |
| **Target GPi** | -1.88 | from -8.23 to 4.46 | 0.56 | 0.7 |
| **MMS** | 0.22 | from -0.92 to 1.35 | 0.71 | 0.3 |
| **Insomnia** | 0.22 | from -1.00 to 1.45 | 0.72 | 0.3 |
| **Hamilton** | -0.045 | from -0.45 to 0.35 | 0.82 | 0.1 |
