# Supplementary material for: Quality of Life after Deep Brain Stimulation in Parkinson's Disease: Does the Target Matter?
Source: Mov Disord Clin Pract. 2024 Sep 3;11(11):1379–87. doi: 10.1002/mdc3.14199 (PMC11542293; doi:10.1002/mdc3.14199)
Supplement: Supplementary file 4 — Data S1. Clinical outcome variables and average stimulation parameters for the Parkinson's disease (PD) cohort. [file MDC3-11-1379-s003.docx]

**Clinical outcome variables and stimulation parameters**

One year postoperatively, patients showed a marked improvement in motor symptoms (MDS-UPDRS-III “off-medication” (39.4%, CI: [33.27, 44.96])), pain score (VAS worst pain (19.5%, CI: [3.74, 32.95])) and dyskinesia scores (Marconi score (65.4%, CI: [54.84, 74.12])) in comparison to the preoperative state. The reduction in LEDD was greater in the STN group (69.8%, CI: [64.00, 74.98]) than in the GPi group (-3.4%, CI: [-30.03, 17.99]). Apathy worsened on average by 20.9%, (CI: [-32.93, -9.84]. All outcome variables are shown in Supplementary Figure 1, and displayed separately by target.

The average parameters for the STN cohort were 2.59 ± 1.04 mA amplitude, 61.1 ± 6.3μs pulse width, and 129.3 ± 14.3 Hz frequency. For GPi patients, the average parameters were 2.92 ± 1.11 mA amplitude, 72.4 ± 33.1 μs pulse width, and 133.6 ± 24.5 Hz frequency.
